# Intolerance in oral versus subcutaneous administration of methotrexate in patients with juvenile idiopathic arthritis: a cross-sectional, observational study

**DOI:** 10.1186/1546-0096-12-S1-P131

**Published:** 2014-09-17

**Authors:** Pieter Van Dijkhuizen, Juliëtte Pouw, Andrea Scheuern, Boris Hügle, Sven Hardt, Gerd Ganser, Jasmin Beate Kümmerle-Deschner, Gerd Horneff, Dirk Holzinger, Maja Bulatović Ćalasan, Nico Wulffraat

**Affiliations:** 1 Paediatric Immunology, Istituto Giannina Gaslini, Genova, Italy; 2 Paediatric Immunology, University Medical Centre Utrecht, Wilhelmina Children's Hospital, Utrecht, Netherlands; 3 Paediatric Immunology, German Centre for Paediatric and Adolescent Rheumatology, Garmisch-Partenkirchen, Germany; 4 Paediatric Immunology, St. Josef-Stift, Sendenhorst, Germany; 5 Paediatric Immunology, University Hospital Tübingen, Tübingen, Germany; 6 Paediatric Immunology, Asklepios Klinik Sankt Augustin, Sankt Augustin, Germany; 7 Paediatric Immunology, University Children’s Hospital Münster, Münster, Germany

## Introduction

Methotrexate (MTX) is the cornerstone disease-modifying anti-rheumatic drug in juvenile idiopathic arthritis (JIA), because of its efficacy. Moreover, serious adverse events are rare. However, in Dutch patients, MTX intolerance, defined as gastro-intestinal side effects occurring not only after, but also before administration of MTX and when thinking of the drug, occurred frequently. MTX intolerance could lead to non-compliance and hence to decreased efficacy, eventually resulting in the necessity to replace it by more costly biologicals. Furthermore, it was observed significantly more often with subcutaneous (SC) than oral (PO) administration, contrary to widely held views. This finding was unexpected and the question about the choice of route of administration when starting MTX in daily clinical practice is still unsolved.

## Objectives

The aim of this study was to assess the prevalence of MTX intolerance and its association with the route of administration in a German cohort of JIA patients to aid physicians in the choice of route of administration.

## Methods

A cross-sectional study of JIA patients on MTX was performed. Primary outcome was intolerance to MTX, which was determined using the validated Methotrexate Intolerance Severity Score (MISS) questionnaire. Clinical and laboratory parameters, among which the route of administration, were compared between the intolerant and tolerant groups in univariate and multivariate analysis.

## Results

Of 179 JIA patients on MTX, 73 (40.8%) were intolerant. Of the 46 patients who received exclusively MTX subcutaneous, 43.5% were intolerant versus 29.5% of 95 who received exclusively MTX PO (P = 0.100). The former experienced significantly more behavioural complaints (76.1% versus 47.4%, P = 0.001) and showed a trend towards more abdominal pain after MTX intake (43.5% versus 27.4%, P = 0.056). Route of administration was associated with MTX intolerance in multivariate analysis, next to the duration of MTX use and the number of active joints.

## Conclusion

The prevalence of MTX intolerance was high. It was higher in patients on MTX SC, but not significantly, and they did experience more adverse effects than those on MTX PO. Therefore, and in the light of other studies in the field demonstrating more adverse effects in patients on MTX SC, PO administration of MTX should be favoured over SC administration when starting MTX.

## Disclosure of interest

None declared.

